# DETERMINANTS AND MAPPING OF LEPTOSPIROSIS IN KEBUMEN, INDONESIA: CASE-CONTROL STUDY

**DOI:** 10.21010/Ajidv19i2.6

**Published:** 2025-04-07

**Authors:** LESTARI Mugi Rahayu, NURCANDRA Fajaria, BUNTARA Arga, SIMANJORANG Chandrayani

**Affiliations:** Department of Public Health, Faculty of Health Science, Universitas Pembangunan Nasional “Veteran” Jakarta, Limo District, Depok City, West Java 16514, Indonesia

**Keywords:** leptospirosis, determinants, risk factors, mapping, case-control, zoonotic, Indonesia

## Abstract

**Background:**

Leptospirosis is a health concern with a high mortality rate. As of 2022, 9.8% of Indonesians are vulnerable to leptospirosis, and Kebumen Regency has been recognized as an endemic area since 2012. This study aims to identify the determinants and map the incidence of leptospirosis in Kebumen, Indonesia, in 2023.

**Material and Methods:**

This study used a 1:1 sample of 53 cases and 53 controls in a case-control study design. Each group was sampled using a purposive sampling technique. Cases were defined as individuals seeking care at a health center or hospital and identified as leptospirosis patients in 2023, while controls were individuals living nearest to the cases. Data analysis for this study involved the use of a logistic regression model.

**Results:**

Individuals with a history of wounds have 40.20 times higher odds of experiencing leptospirosis. Men have 2.58 times higher odds of experiencing leptospirosis, while poor use of personal protective equipment (PPE) increases the odds by 2.27. Leptospirosis risk is elevated in areas where rats and standing water are prevalent, as these factors are typically found nearby. Furthermore, pets at risk are commonly found in high-risk areas.

**Conclusion:**

History of wounds, sex, and use of PPE are factors that can predict the incidence of leptospirosis. Environmental determinant mapping is observed in high-density, geographically proximate locations.

## Introduction

Leptospirosis is one of the infectious diseases with high mortality. Many tropical regions are home to the common and possibly lethal zoonotic illness leptospirosis. Severe rainstorms and flooding are frequently linked to significant outbreaks (Haake and Levett, 2015; Lau et al., 2018; Sokolova et al., 2021). *Leptospira spp*. are the pathogenic germs that cause leptospirosis and can spread directly or indirectly from animals to people (Centers for Disease Control and Prevention (CDC), 2024; World Health Organization (WHO), 2003). The majority of leptospirosis cases that are documented have severe symptoms, and between 5% and 15% of cases are fatal (Centers for Disease Control and Prevention (CDC), 2018). The case fatality rate for patients suffering from severe pulmonary hemorrhage syndrome can surpass 50% (Centers for Disease Control and Prevention (CDC), 2018; Marotto et al., 1999; Nicodemo et al., 1997; Panaphut et al., 2002; Yersin et al., 2000). Clinical signs might range from minor illnesses to serious diseases (like Weil’s disease) (Levett, 2001; The Center For Food Security and Public Health, 2013).

Wounds, abrasions, or mucous membranes are portals of entry for infection. High-risk individuals include veterinarians, slaughterhouse workers, ranch workers, hunters, animal rescue workers, scientists, and animal technologists in the laboratory or field (Steneroden et al., 2011). Furthermore, indirect contact with soil or water exposed to leptospira is more frequent. Activities related to work, play, or hobbies can be connected to this. Gardeners and agricultural laborers are susceptible to leptospirosis (Levett in Haake and Levett, 2015).

Based on estimates from Costa et al. (2015), leptospirosis is thought to be responsible for 1.03 million infections and 58,900 deaths globally per year. Leptospirosis is expected to cause 290 million Disability Adjusted Life Years (DALYs) and 1.25—4.54 million Uncertainty Intervals (UI) annually per country. Included in this figure are 280 million Years of Life Lost (YLL) (1.16 million–4.46 million) and 103,200 (38,800—188,100) Years of Life with Disability (YLD). The corresponding incidence is 41.8 DALYs per 100,000 persons per year (UI 18.1—65.5) (Torgerson et al., 2015).

In 2022, DKI Jakarta, West Java, Central Java, DI Yogyakarta, East Java, Banten, North Kalimantan, South Sulawesi, Southeast Sulawesi, and East Kalimantan reported 1,419 leptospirosis cases. Based on the number of cases, there were 139 fatalities, resulting in a Case Fatality Rate (CFR) of 9.8%. Compared to 2021, leptospirosis cases increased from 734 (CFR 11.4%) to 1,419 (CFR 9.8%) in 2022. Central Java Province was responsible for 35.4% of leptospirosis cases, followed by East Java with 28.3% (Ministry of Health of the Republic of Indonesia, 2022). Kebumen is one of the districts in Central Java with the highest prevalence of leptospirosis, with cases reported annually. According to the Health and Population and Family Planning Office of Kebumen Regency, the number of leptospirosis cases grew by 23 cases (CFR 39.1%) in 2021, 62 cases (CFR 14.5%) in 2022, and 137 cases in 2023 (CFR 13.9%).

According to the data presented above, leptospirosis is a public health concern in Indonesia, particularly in Kebumen, one of the endemic areas (Center for Environmental Health Engineering and Disease Control Yogyakarta, 2019; Health and Population and Family Planning Office of Kebumen Regency, 2023). Research on risk factors associated with individual, environmental, and behavioral factors has not been conducted concurrently in Kebumen. Furthermore, existing prevention efforts have proven inadequate as they tend to focus primarily on behavioral characteristics, and public awareness remains insufficient. Consequently, this study is crucial for identifying the determinants related to the incidence of leptospirosis and for mapping the environmental risk factors associated with its incidence in Kebumen. The findings of this study are intended to inform the development of effective leptospirosis prevention policies.

## Methods

### Research design and study population

This quantitative study of an unmatched case-control approach was performed to identify the determinants of leptospirosis and map its incidence in Kebumen for 2023. Conducted from January to June 2024 in Kebumen Regency, Central Java, Indonesia, the study population included all individuals residing in Kebumen Regency during 2023.

The sample was organized into case and control groups, selected through purposive sampling in the Kuwarasan, Buayan, Sruweng, and Adimulyo districts, which were identified as areas with the highest incidence of cases. Overall, the study comprised 53 cases and 53 controls. Cases were defined as individuals seeking treatment at a health center in Kebumen Regency and receiving a leptospirosis diagnosis. At the same time, the controls were neighbors of the cases who had never been diagnosed with leptospirosis. No variables were matched in this study.

### Study instrument and study variables

The study examined several variables: age, gender, education, occupation, history of injury, rats, puddles, pets, personal protective equipment (PPE), food storage practices, and personal hygiene. However, variables related to contact with puddles and the habit of bathing or washing in rivers were excluded from the analysis, as validity and reliability tests indicated that the associated questions were neither valid nor reliable. The researchers discovered that the characteristics of the subjects regarding these variables were largely homogeneous. In fact, 90% of the subjects never used puddles or rivers. They chose to go home and bathed at home since there were no puddles and rivers around them.

Data collection was carried out using a semi-structured questionnaire organized into seven distinct sections:

1. individual characteristics: this section gathered details such as name, date of birth, gender, education, occupation, and medical history;
2. history of injury during the outbreak;
3. presence of rats during the outbreak: questions in this section addressed both the presence of rats and the respondents’ behaviors in managing this issue;
4. presence of puddles during the outbreak;
5. presence of pets during the outbreak: this part focused on the presence of pets and the respondents’ approaches to handling them;
6. behavior during and after work during the outbreak and
7. food and drink storage practices during the outbreak.

### Validation test of the questionnaire

Validity and reliability tests were conducted in Samping Village, located in Kemiri District, Purworejo Regency. This location was selected due to the similarity in characteristics among the research subjects. Purworejo Regency and Kebumen Regency both feature a combination of rice fields and livestock farming in terms of environmental factors. Additionally, their demographic factors, including work and education, are quite similar. These parallels suggest that the habits and behaviors of the residents in both regions are alike. Historically, both regencies were called Kedu Residency which was recognized since Dutch East Indies colonial administration due to similar culture, behavior, topography, and languages. The Pearson product moment correlation validity test was performed through direct interviews utilizing a prepared questionnaire. A total of 20 respondents participated in the validity and reliability assessment of the questionnaire. For the validity test, an α value of 0.05 and an r-table value of 0.44 were employed. The results indicated that eight variables were valid (with r-count > r-table). In comparison, two variables—concerning contact with puddles and the habits of bathing or washing in rivers—were deemed invalid (with r-count < r-table). Additionally, for the eight valid variables, Cronbach’s Alpha value of ≥ 0.6 was achieved, confirming the reliability of the questionnaire.

### Data collection

Data collection took place in May 2024. The number of leptospirosis cases in each sub-district was sourced from the Health and Population and Family Planning Control Office of Kebumen Regency. Additionally, the names and addresses of leptospirosis patients were obtained from health centers in the Kuwarasan, Buayan, Sruweng, and Adimulyo Districts, which reported the highest incidence of cases. The data were gathered directly from respondents ’ homes using a semi-structured questionnaire. Subject data and information were kept confidential and used only for this research. All respondents provided informed consent before the interview and geographical data collection. The coordinate points used were the locations of households in the case group.

### Statistical analysis

Chi-square tests and simple logistic regression were conducted to assess the potential of various variables for multivariate analysis. Multiple logistic regression was utilized to identify the most effective model for explaining the incidence of leptospirosis. QGIS with merge analysis was also employed to map the environmental risk factors associated with leptospirosis patients in Kebumen.

## Results

### Overview of leptospirosis incidents in Kebumen in 2023

Based on data from the Health and Population and Family Planning Office of Kebumen Regency, there were 137 cases and 19 deaths (13.9% CFR) due to leptospirosis in 2023 in Kebumen, Central Java, Indonesia.

**Figure 1:**
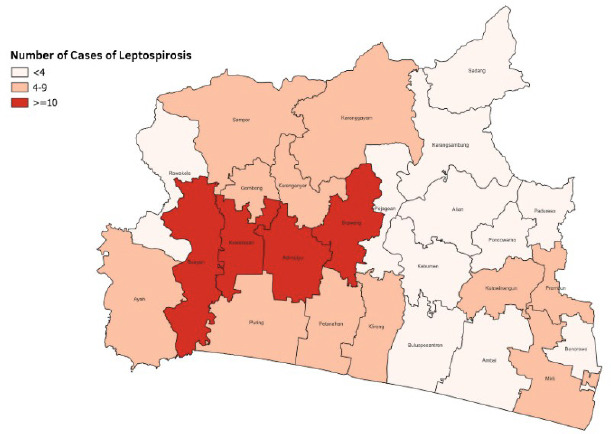
Number of Cases of Leptospirosis in Kebumen, Indonesia

Figure 1 shows that the darker color on the map indicates higher positive cases of leptospirosis. The locations with the highest cases were Sruweng, Adimulyo, Kuwarasan, and Buayan Districts. The figure also demonstrates that the locations with the most cases were adjacent. Furthermore, data from the Health and Population and Family Planning Office of Kebumen Regency revealed that the highest number of deaths were found in Kuwarasan District, totaling six deaths. Based on the four districts with the highest incidence of leptospirosis, the distribution of cases based on the month of illness is detailed in Table 1.

**Table 1:** Distribution of leptospirosis cases based on month of illness

District	Month						Total

	Jan	Feb	Mar	Apr	May	Jun	
Adimulyo	0	1	5	2	0	0	8
Buayan	1	4	10	0	0	0	15
Kuwarasan	0	3	11	2	0	1	17
Sruweng	0	1	10	1	1	0	13

**Total**	1	9	36	5	1	1	53

Table 1 confirms the incidence of leptospirosis in Kebumen, Central Java, Indonesia, in 2023. The first case was reported in the Buayan Sub-district. The peak number of cases was 36 in March. The cases decreased dramatically in the following months, concluding with June in the Kuwarasan Sub-district.

### Determinants of leptospirosis

Data were obtained from 53 cases and 53 controls. After categorization with ROC value, the cut-off point for leptospirosis patients was 45 years of age or older (Table 2).

**Table 2:** Individual determinants of leptospirosis

Variable	Leptospirosis				Total		p-value	OR crude (95% CI)

	Case		Control					

	n	%	n	%	n	%		
Age								
≥45	27	50.94	26	49.06	53	50.00	0.85	1,08 (0,47—2,48)
<45	26	49.06	27	50.94	53	50.00		Ref
Sex								
Male	47	88.68	32	60.38	79	74.53	0.00	5,14 (1,73—17,08)
Female	6	11.32	21	39.62	27	25.47		Ref
Education								
≤Elementary sch	32	60.38	31	58.49	63	59.43	0.67	1,24 (0,47—3,28)
Junior high sch	11	20.75	10	18.87	21	19.81	0.65	1,32 (0,40—4,38)
≥Senior high sch	10	18.87	12	22.64	22	20.75		Ref
Occupation								
Farmer	47	88.68	30	56.60	77	72.64	0.00	6,58 (2,24—19,33)
Planter	1	1.89	2	3.77	3	2.83	0.58	2,1 (0,16—28,02)
Others	5	9.43	21	39.62	26	24.53		Ref
Wound history								
Yes	27	50.94	1	1.89	28	26.42	0.00	54,00 (7,75—2251,60)
No	26	49.06	52	98.11	78	73.58		Ref

Leptospirosis sufferers were predominantly aged 45 years or older, constituting 50.94% of the cases, while the control group had a higher percentage of individuals aged younger than 45 years (also 50.94%). In terms of sex, 88.68% of the cases were male, a figure that exceeded that of the controls. The chi-square analysis indicated a significant association between gender and the incidence of leptospirosis (p-value = 0.00). The odds ratio (OR) calculated was 5.14, with a 95% CI of 1.73—17.08. This suggests that men have 5.14 times the odds of suffering from leptospirosis compared to women.

Furthermore, 60.38% of leptospirosis patients had an education level of elementary school or lower, which was higher than the proportion observed in the control group. Regarding occupation, 88.68% of those afflicted worked as farmers, much more prevalent than the controls. The chi-square test revealed a significant relationship between farming and the incidence of leptospirosis (p-value = 0.00), whereas the planters category showed no significant association (p-value = 0.58). In this context, farmers had 6.58 times the odds of contracting leptospirosis compared to individuals in other occupations (95% CI: 2.24-19.33).

Additionally, 50.94% of leptospirosis patients reported a history of wounds on their hands or feet, a figure that was higher than that of the controls. The chi-square analysis also indicated a significant correlation between wound history and the incidence of leptospirosis (p-value = 0.00). Individuals with a history of wounds at the time of the leptospirosis outbreak in Kebumen had 54 times greater odds of developing the disease compared to those without such a history.

Table 3 illustrates that both individuals suffering from leptospirosis and those who did not were similarly exposed to rats in their home environments, with a prevalence of 92.45%. The results from the chi-square analysis indicate that the presence of rats has a p-value of 1.00, suggesting no significant association between rats and the incidence of leptospirosis. Regarding the presence of puddles in the surrounding environment, it was observed that the proportion of leptospirosis cases (54.72%) is lower than that of the control group. When considering the presence of pets, 67.92% of leptospirosis cases were found to have pets, which is a smaller proportion than the control group. The chi-square analysis shows that both the presence of puddles (p-value = 0.43) and the presence of pets (p-value = 0.19) lack a significant relationship with the occurrence of leptospirosis.

**Table 3:** Environmental determinants of leptospirosis

Variable	Leptospirosis				Total		p-value	OR crude (95% CI)

	Case		Control					

	n	%	n	%	n	%		
Rats present								
Present	49	92.45	49	92.45	98	92.45	1.00	1,00 (0,18—5,60)
None	4	7.55	4	7.55	8	7.55		Ref
Water puddle								
Present	29	54.72	33	62.26	62	58.49	0.43	1,37 (0,59—3,19)
None	24	45.28	20	37.74	44	41.51		Ref
Pet present								
Present	36	67.92	42	79.25	78	73.58	0.19	0,55 (0,21—1,45)
None	17	32.08	11	20.75	28	26.42		Ref

Table 4 indicates that patients with leptospirosis exhibit a higher proportion of inadequate personal protective equipment (PPE) use, at 84.91%, compared to controls. The chi-square analysis results reveal a significant relationship between PPE use and the incidence of leptospirosis (p-value = 0.01). Individuals with poor PPE usage have 3.41 times greater odds of contracting leptospirosis than those who use PPE adequately. Regarding personal hygiene, the case group reports a commendable level of hygiene at 93.34%, although this is still lower than the control group. Regarding food storage practices, the proportion of closed food storage among the case group is smaller than that of the control group, with a case proportion of 96.23%. The chi-square analysis for personal hygiene (p-value = 0.31) and food storage conditions (p-value = 0.56) indicates that neither variable has a significant relationship with the incidence of leptospirosis.

**Table 4:** Behavioral determinants of leptospirosis

Variable	Leptospirosis				Total		p-value	Crude OR (95% CI)

	Case		Control					

	n	%	n	%	n	%		
PPE use								
Poor	45	84.91	33	62.26	78	73.58	0.01	3,41 (1,24—9,99)
Good	8	15.09	20	37.74	28	26.42		Ref
Personal hygiene								
Poor	3	5.66	1	1.89	4	3.77	0.31	3,12 (0,24—166,87)
Good	50	93.34	52	98.11	102	96.23		Ref
Food storage								
Poor	2	3.77	1	1.89	3	2.83	0.56	2,04 (0,10—122,57)
Good	51	96.23	52	98.11	103	97.17		Ref

Table 5 shows the p-value logistic regression analysis of the model conducted through the LR test yielded a value of 0.05, indicating a significant multivariate analysis fit model. The final modeling results revealed that the determinants associated with leptospirosis included a history of wounds, sex, and use of PPE. Among these determinants, the history of wounds emerged as the most dominant variable influencing the incidence of leptospirosis, with an adjusted odds ratio (OR) of 40.20 (95% CI: 5.05—320.03). This suggests that individuals with a history of wounds have 40.2 times higher odds of experiencing leptospirosis compared to those without such a history. Additionally, the analysis indicated that sex was the second most influential variable in the incidence of leptospirosis, with an adjusted OR of 2.58 (95% CI: 0.85—7.82). This signifies that males have 2.58 times higher odds of experiencing leptospirosis than females. Lastly, using personal protective equipment (PPE) was identified as another variable influencing the incidence of leptospirosis. The adjusted OR for the use of PPE was 2.27 (95% CI: 0.76—6.78), indicating that individuals with poor PPE usage have 2.27 times higher odds of experiencing leptospirosis compared to those with good PPE usage.

**Table 5:** Final model of leptospirosis determinants

Variable	p-value	aOR (95% CI%)
Wound history	0.00	40.20 (5.05—320.03)
Male	0.09	2.58 (0.85—7.82)
Poor PPE use	0.14	2.27 (0.76—6.78)

### Mapping leptospirosis

This mapping aimed to outline areas with the highest risk based on the presence of rats, puddles, and pets.

**Figure 2:**
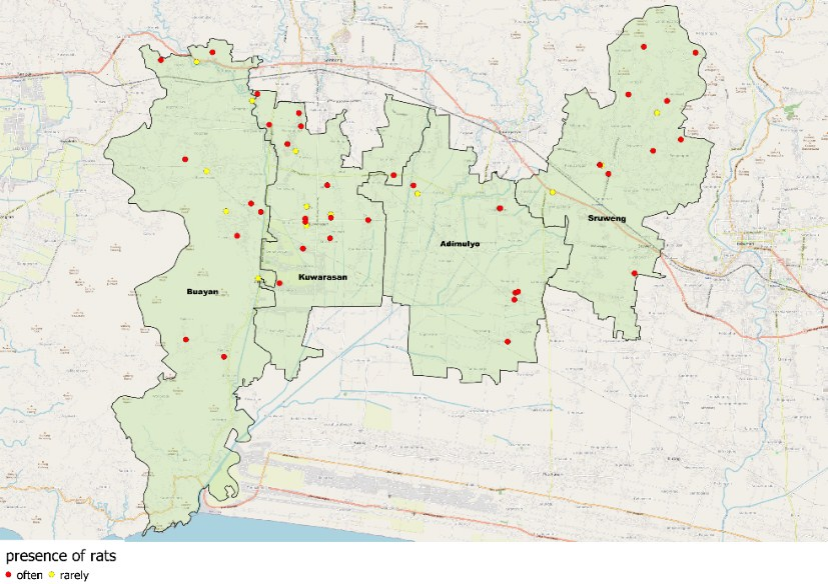
Map of rat distribution based on leptospirosis cases in Kebumen in 2023

Figure 2 illustrates that rats are most frequently found in the Kuwarasan District. The frequency of rat sightings is notably high at adjacent points, particularly in the Kuwarasan and Buayan Districts.

**Figure 3:**
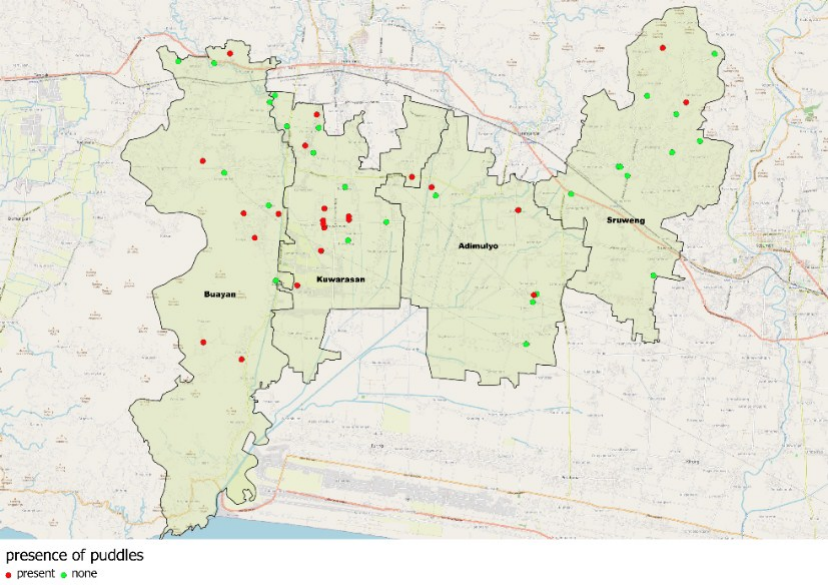
Map of water stagnation distribution based on leptospirosis cases in Kebumen in 2023

Figure 3 reveals that points with puddles tend to be clustered in adjacent areas, as seen in the Kuwarasan and Buayan Districts. The Kuwarasan District has the highest number of case points with puddles in the home environment.

**Figure 4:**
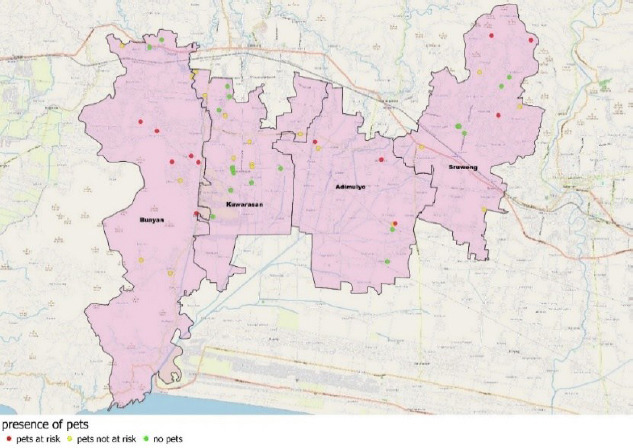
Map of pet distribution based on leptospirosis cases in Kebumen in 2023

Figure 4 displays the distribution of pets most at risk in the Buayan District compared to the conditions in the Kuwarasan District. In Kuwarasan, there were no cases of at-risk pets in the surrounding environment.

## Discussion

### Environmental Risk Factor Mapping

The highest frequency of rats in the distribution results was in Kuwarasan District, which could be associated with the large area of rice fields there. Kuwarasan District has 62.61% of the total area of the district (Badan Pusat Statistik (BPS) Kabupaten Kebumen, 2019). Based on the distribution of water puddles, Kuwarasan District and Buayan District had close and relatively many case points. Based on the BPS Kecamatan Buayan (2019), the amount of rainfall in January 2018 was 463 mm, and the lowest was in June, with a rainfall of 34 mm. For Kuwarasan District, the rainfall was 4,183 mm with 123 rainy days (Badan Pusat Statistik (BPS) Kabupaten Kebumen, 2019). Puddles were created and flooding was caused by heavy rainfall (Rahayu et al., 2018). After heavy rain or flooding, puddles of muck and water could provide a home for *Leptospira* bacteria (Damalia et al., 2021). Water puddles on the ground surface surrounding the house had the potential to be accessible to rats and passed by people. This theory was supported by Yanagihara et al. (2022), which showed that the number of pathogenic and saprophytic leptospira increased in wet soil but not in water or soil alone. Furthermore, leptospira can proliferate in an environment with rising water levels since they are in a latent stage in the soil. The discovery that leptospirosis cases are substantially greater during the rainy season and rise following floods provides strong support for this theory.

Based on the distribution of pets, pet ownership was at greater risk in the Buayan District, where 55% of village areas are highlands. Pet or cattle ownership was at risk, particularly concerning cows and goats. Cows could release significant quantities of Leptospira, averaging 6.3 × 10^8^ per day (Barragan et al., 2017). Additionally, sheep and goats are capable of excreting *L. interrogans* in their urine, thereby facilitating transmission to other animals and humans (Hajikolaei et al., 2022). Sunaryo and Priyanto’s (2022) research in the Bantul and Gunungkidul Regencies revealed a relatively high prevalence of Leptospira in livestock. In Bantul Regency, the prevalence in cattle was found to be 63.64%, whereas in goats and sheep, it was recorded at 22.22%. Additionally, findings from Gunungkidul Regency indicated a prevalence of 50.00% in cattle and 45.16% in goats and sheep. Furthermore, research by Widiasih et al. (2021) in Kulon Progo highlighted that many farmers still lack adequate knowledge about Leptospira. This is an important issue that needs to be addressed to ensure effective preventive measures are implemented.

### Wound History Variable

People with a history of wounds had 40.20 higher odds of experiencing leptospirosis than people without wounds (95% CI: 5.05—320.03). Almost all leptospirosis sufferers with a history of wounds (95.83%) did not cover the wounds and worked in the rice fields. It is known that the entry point for leptospirosis can be through wounds, abrasions, mucous membranes, or through waterlogged skin. If someone with wounds interacts with the urine of rats or other animals infected with leptospirosis, leptospira bacteria will enter and infect through the wound (Ariani and Wahyono, 2020; Steneroden et al., 2011; World Health Organization (WHO), 2003).

Cahyati and Lestari’s (2009) research found that someone with a history of wounds has a 6 times higher risk of experiencing leptospirosis compared to people who do not have a history of wounds (p-value = 0.027). Sofiyani et al. (2018) found that a history of wounds can increase the likelihood of someone experiencing leptospirosis by a 1.98 higher risk (p-value = 0.008; 95% CI: 0.40—2.87). In addition, research by Pratamawati et al. (2018) stated that covering an open wound with a plaster protects against leptospirosis. Individuals who cover their wounds are 0.12 times less likely to contract leptospirosis than those who leave uncovered (95% CI: 0.03—0.51).

### Sex Variable

Men had 2.58 higher odds of experiencing leptospirosis than women (p-value = 0.09; 95% CI: 0.85—7.82). This agrees with the study by Delight et al. (2024), which found that men have 1.76 higher odds of leptospira seropositivity (95% CI 1.11—2.79) than women. The relationship between men and leptospirosis is explained by the fact that men are usually more exposed to water and are more likely to engage in risky work or recreational activities. Where this is a predisposition to leptospirosis (Skufca and Arima, 2012; Tomizawa et al., 2017). Although gender in this study was not statistically associated, the role of gender in the incidence of leptospirosis in this population was important. This is due to the majority of the population in this study being male farmers. Male farmers also tend to work in rice fields more often than female farmers, who are likely to be contaminated with *Leptospira sp* from rice field rats. A study by Caruso et al. (2013) supports that there are differences in the immune response to Leptospira in men and women. Sexual dimorphism in the immune response means that women are more resistant to infection. Research by Guerra-Silveira and Abad-Franch (2013) suggests that the possibility of certain genetic factors that can determine differences in immune responses between men and women may be linked to the X chromosome. This is because it is known that several genes involved in immunity are located on this chromosome (Guerra-Silveira and Abad-Franch, 2013; Skufca and Arima, 2012). Since male farmers were found to be more at risk than female farmers, interventions and education for leptospirosis prevention could be targeted at male farmers to avoid another leptospirosis outbreak in the future in this population.

### PPE Usage Variable

The use of PPE is a risk factor for leptospirosis. The results showed that poor use of PPE could increase the odds of someone experiencing leptospirosis by 2.27 higher than the good use of PPE (p-value = 0.14; 95% CI:0.76—6.78). Those considered to use the PPE poorly were ones who used PPE (gloves, boots, long pants, and long shirts) less than or equal to two, while good use of PPE was more than 2 uses of PPE when doing work. The use of PPE while working in rice fields was not found to be statistically significant, but PPE plays an important role in protecting farmers from leptospirosis infection. Especially when farmers have wounds on their feet and hands. The role of PPE in protecting various diseases has been widely confirmed and this study cannot ignore it even though it was not statistically significant. It could be the small sample size of this study that caused the insignificance. If the sample is increased by about two or three times, it is possible that the confidence interval will narrow and will not pass the null value so that it will be statistically significant.

Research by Sofiyani et al. (2018) showed that the use of PPE also reduces the likelihood of someone getting injured by 2.54 lower (p-value = <0.001, 95% CI: -3,49— -1,60). This study shows that using PPE aligns with the reduced likelihood of wounds appearing, reducing the possibility of someone experiencing leptospirosis. Ginting and Indiarjo (2022) research shows the results of the analysis of the use of each PPE. The results show that the use of gloves and footwear has a significant relationship with the occurrence of leptospirosis. A person who does not wear gloves has a 4.34 higher odds of experiencing leptospirosis than someone who wears gloves (p-value = 0.00; 95% CI: 1.60—11.78). In addition, a person who does not wear footwear increases the odds of experiencing leptospirosis by 3.20 higher than someone who wears footwear (p-value = 0.01; 95% CI: 1.25—8.17). The results showed that long pants and long shirts did not have a significant relationship with p-values of 0.81 and 0.66, respectively. Proper use of PPE by farmers can protect them from occupational exposure not only from pesticide exposure, but also from the risk of leptospirosis infection (Nurcandra et al., 2018).

### Research limitations

This study presents several limitations, including 1) recall bias concerning variables that depend on respondents’ memories due to past exposure, so that risk estimates can be distorted or less accurate. To minimize this bias, researchers designed research questions by specifying the exposure period; 2) sharing exposure bias related to environmental risk factors, which obscured their connection to leptospirosis incidence—this occurs because the control group was selected from nearby neighborhoods, where it was assumed that similar exposed variables conditions led to the same risk of developing the disease; 3) non-response rate that led to an underestimation of research results, with a 5.36% reduction in cases and 3.64% in controls. Fortunately, the non-response rate of this study was low. Based on this condition, we suggest that future researchers should control this bias by considering the length of data collection and preparing communications that can reach people with disabilities; 4) inadequate measurement of environmental variables, where the assessment of rat presence was not based on successful capture methods (success trap), the evaluation of puddles did not consider the permanent water bodies, and the measurement of pet presence is not constrained by type; and 5) The chance probably existed in wound history variables. The treatment received by both case and control groups was the same during data collection. Thus, a nondifferential misclassification occurred and caused the results of this study to be underestimated.

## Conclusion

In Kebumen, leptospirosis was reported to have caused 137 cases and 19 fatalities in 2023 (CFR 13.9%). The leptospirosis incident happened between January and June of 2023, with March of that year seeing the highest number of cases. Adimulyo Subdistrict, Sruweng Subdistrict, Buayan Subdistrict, and Kuwarasan Subdistrict were the subdistricts with the highest number of leptospirosis patients.

The results of mapping environmental risk variables show that the locations of at-risk water puddles and the presence of rats were typically near each other. Pets at greatest risk were in areas that tend to be geographically elevated. Modeling using factors for wound history, sex, and PPE use can forecast the incidence of leptospirosis in Kebumen in 2023.

## Ethics approval

The Commission of the Health Research Ethics of the Faculty of Public Health, University of Muhammadiyah Jakarta (UMJ) approved the study on May 6, 2024, with the ethics number 10.105.B/KEPK-FKMUMJ/V/2024.

## Competing interests

All the authors declare that there are no conflicts of interest.

**List of Abbreviations:**
aOR: Adjusted Odds Ratio,
CDC: Centers for Disease Control and Prevention,
CFR: Case Fatality Rate,
CI: Confidence Interval,
DALYs: Disability Adjusted Life Years,
GIS: Geographic Information System,
OR: Odds Ratio,
PPE: Personal Protective Equipment,
UI: Uncertainty Intervals,
WHO: World Health Organization,
YLD: Years of Life with Disability,
YLL: Years of Life Lost.
